# The Value Surgical Services Bring to Critical Access Hospitals

**DOI:** 10.7759/cureus.14367

**Published:** 2021-04-08

**Authors:** Nathanael N Hoskins, Marco A Cunicelli, Wade Hopper, Robert Zeller, Ning Cheng, Tom Lindsey

**Affiliations:** 1 Surgery, Edward Via College of Osteopathic Medicine, Blacksburg, USA; 2 Surgery, Edward Via College of Osteopathic Medicine, Spartanburg, USA; 3 Biostatistics, Edward Via College of Osteopathic Medicine, Auburn, USA

**Keywords:** critical access hospital, hospital profitability, net income, hospital viability, rural surgery, rural hospital, hospital sustainability

## Abstract

Purpose

Critical Access Hospitals (CAHs) serve rural populations and receive government subsidies to compensate for their relatively high overhead costs and low occupancy rates. Twenty-nine percent of all hospitalizations in the United States include a surgical procedure, and hospitalizations involving surgery accounted for nearly half of all hospital revenue in 2011. This study aims to determine the value surgical services bring to CAHs and their impact on the viability of these facilities.

Methods

Public access data from the American Hospital Directory (AHD) was analyzed about each hospital's revenue and surgical services offered. Excel was utilized to randomly select 300 CAHs from a pool of 1350 CAHs based on a 95% confidence interval and a 5% margin of error. Linear regression models were fit to the data evaluating the association of net income with the number of surgical services offered per hospital and the association of total margin with the number of surgical services offered per hospital. Models were adjusted for location, occupancy rate, and case mix index.

Findings

The linear regression model demonstrated that for every additional surgical service provided by a CAH, the hospital net income increased by $630,528 (p=0.0032). A similar trend was observed when modeling profitability. The total margin increased 0.73% for each additional surgical service added, albeit without statistical significance (p=0.1342). CAHs providing two or three surgical services showed tighter group variance than those not offering surgery or only offering one surgical service.

Conclusions

Net income was significantly correlated to the number of surgical services offered at CAHs. Furthermore, CAHs offering more surgical services seem to have more predictable profits than those offering less surgical services. CAHs would financially benefit from offering more or expanding surgical services at their facilities.

## Introduction

In 1997, the United States federal government created the Critical Access Hospital (CAH) program as part of the Balanced Budget Act [1]. This program was designed to support rural hospitals which serve populations lacking healthcare availability and emergency services by increasing financial incentives from the federal government. CAHs must meet several requirements to qualify for federal funding: they must have 25 beds or less, offer 24/7 emergency care, and be located more than 35 miles from another hospital. The "Critical Access Hospital” designation provides significant financial supplementation for hospitals through guaranteed mortgages, access to pre-allocated grant money, increased Medicare reimbursement with a payout rate of 101%, and inclusion in the 340B Drug Pricing Program [1]. Evidence shows these financial incentives have helped sustain profitability and quality of care at CAHs. From 2011 to 2017, nonprofit rural CAHs were shown to be more profitable than rural hospitals lacking the CAH designation. Additionally, studies have concluded that qualitative safety outcomes at CAHs are generally equivalent to those at non-CAHs [2-5].

Since 2005 there have been 170 rural hospital closures, with 128 of these occurring within the last 10 years [6]. Simultaneously, between 2001 and 2008 there has also been a decrease in surgical services offered by rural hospitals, with the largest decrease occurring in CAHs [7]. However, there is evidence of a correlation in the degree of rurality and increased need for surgical services among a variety of procedures for Medicare patients [8].

It is well known that surgeries contribute greatly to hospital profits, however, not all CAHs provide surgical services [9]. Twenty-nine percent of all hospitalizations in the United States include a surgical procedure. Furthermore, hospitalizations that involved surgery accounted for nearly half of all hospital revenue in 2011. Prior studies have analyzed the availability of surgical services in CAHs. In 2011, 87% of CAHs had surgical capabilities [10]. Despite this, CAHs reported lower surgical volumes and surgical Medicare profits compared to non-CAH rural hospitals [7]. In a survey of CAH administrators, 81% reported that the provision of surgery was “very important” to their hospital’s viability; additionally, 12% claimed their hospital “would close” if its surgical capacities were to be lost [11].

Previous studies have demonstrated a direct correlation between the profitability of rural hospitals and their surgical volume [12,13]. Presently, no study has analyzed the relationship between profit and the number of surgical services offered specifically at CAHs. This study aims to quantify the value that surgical services bring to CAHs, as this could provide compelling evidence to increase the number of surgical services offered at CAHs.

## Materials and methods

A cross-sectional study was conducted on hospital cost using a secondary data analysis of publicly reported data by the American Hospital Directory (AHD) in August 2020. AHD Inc. is a private company that amasses data from more than 7,000 hospitals nationwide. Both public and private data including Medicare claims, hospital cost reports, and commercial licensors are included [14]. These data were collected from each hospital’s most recent Medicare Cost Report as reported to the Center for Medicare and Medicaid Services’ (CMS) Healthcare Cost Report Information System (HCRIS) [14]. The AHD compiles these data and creates a publicly searchable database.

Using a 95% confidence interval (z=1.96, e=0.05), 80% power, α=0.05, population proportion of 50% (p=0.5), and a population size (N) of 1350 total CAHs in the US, it was determined a sample size (n) of 300 would sufficiently represent all CAHs. Next, 300 out of the 1350 CAHs in the U.S were randomly selected for the study using a randomization formula in Excel (Microsoft, Redmond, WA, USA) [15].

The independent variable was the number of surgical services offered at each hospital. The American College of Surgeons recognizes 14 surgical specialties: cardiothoracic surgery, colon and rectal surgery, general surgery, gynecology and obstetrics, gynecologic oncology, neurological surgery, ophthalmic surgery, oral and maxillofacial surgery, orthopedic surgery, otorhinolaryngology, pediatric surgery, plastic and maxillofacial surgery, urology, and vascular surgery [16]. Surgical services were defined as above for this paper. However, only orthopedic surgery, general surgery, cardiovascular surgery, vascular surgery, urology, and neurosurgery were observed within the sample. Of note, some CAHs offered both orthopedics and orthopedic surgery. In these cases, orthopedic surgery data was utilized while orthopedics data were excluded from the study as orthopedics did not include surgical procedures.

There were two outcome variables: net income and total margin. Net income was defined as total revenue minus total expenses. Total margin was defined as total revenue minus total expenses, divided by total revenue. Net income reflects the profit of a given hospital, whereas total margin reflects the profitability. Profit is an absolute amount of money, whereas profitability is a measure of profit relative to baseline costs. Using the raw data, a mean and standard error were calculated for both net income and total margin for all sampled CAHs (n=300) and again for the CAHs offering surgical services (n=200).

**Figure 1:**
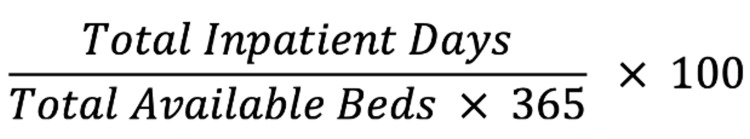
Occupancy Rate Formula

The two primary outcome variables were adjusted using three co-variates extracted from the AHD data: case mix index (CMI), occupancy rate (OR), and geographic region. The CMI is a hospital’s one-year average Diagnostic-Related Group weight which reflects the average case complexity that a hospital sees over the course of one year [17]. Occupancy Rate was calculated as the percent of a hospital’s beds that were occupied over the course of the reported year according to Figure 1. Finally, each CAH was assigned a geographic region of the United States (Northeast, South, West, or Midwest) consistent with regions defined by the U.S. Census Bureau [18].

To evaluate profitability relative to the presence of surgical services, a linear regression model was used to evaluate the association of the dependent variable with the independent variable of surgical services offered per CAH. The dependent variables were net income and total margin. A P-value of ≤ 0.05 was set as the significance threshold. In the linear regression model, the intercept parameter was defined as the predicted value for the model when all other parameters were set to zero. The parameter estimate was defined as the change in the dependent variable according to every one-unit change per predictor variable while all other predictors were kept constant. Data were stored in a Microsoft Excel spreadsheet and analysis was performed by Statistical Analysis System (SAS) 9.4 (SAS Institute Inc., Cary, NC, USA).

## Results

Among all 300 sampled CAHs, the mean net income was $954,204 and the mean total margin was 0.94% (Table 1). CAHs without surgery programs (n=100) demonstrated a mean total margin of -0.41%, while CAHs with surgery programs (n=200) showed a mean total margin of 1.61%. The co-variable analysis showed an average CMI of 1.06 as well as an average occupancy rate of 37.64%. Furthermore, the regional analysis of the sample yielded the following distribution: 127 CAHs were considered Midwestern (42.5%), 92 CAHs were Southern (30.8%), 61 CAHs were Western (20.4%), and 19 CAHs were Northeastern (6.3%) (Table 1). Out of 300 CAHs, 200 offered one or more surgical services (Table 2). Among CAHs that offered surgical services, the most common specialty offered was urology (97%), followed by orthopedic surgery (36%) and then general surgery (22%) (Table 3).

**Table 1:** Descriptive Statistics of Raw Data CAH: Critical Access Hospital

Variable	Mean ± SE
Number of Surgical Services	1.04 ± 0.06
Net Income – All sampled CAHs	$954,204 ± $172,324
Total Margin - All sampled CAHs	0.94% ± 0.39%
Total Margin – CAHs with surgical services (n=200)	1.61% ± 0.39%
Total Margin – CAHs without surgical services (n=100)	-0.41% ± 0.85%
Occupancy Rate	37.64% ± 1.27%
Case Mix Index (CMI)	1.06 ± 0.01
Region - West	61 CAHs (20.4%)
Region - Midwest	127 CAHs (42.5%)
Region - South	92 CAHs (30.8%)
Region - Northeast	19 CAHs (6.3%)

**Table 2:** Number of Surgical Services Offered at Critical Access Hospitals Among 300 Critical Access Hospitals sampled, one-third (n=100) offered no surgical services.

Number of Surgical Services	Critical Access Hospitals (n = 300)
0	100 (33%)
1	126 (42%)
2	42 (14%)
3	32 (11%)
4+	1 (<1%)

**Table 3:** Most Common Types of Surgical Services Offered at Critical Access Hospitals Out of 200 Critical Access Hospitals providing surgical care, 97% (n=194) offered urologic surgical services, 26% (n=71) offered orthopedic surgical services, and 22% (n=44) offered general surgical services.

Surgical Specialty	Hospitals offering surgery (n = 200)
General Surgery	44 (22%)
Orthopedic Surgery	71 (36%)
Urology	194 (71%)

**Figure 2:**
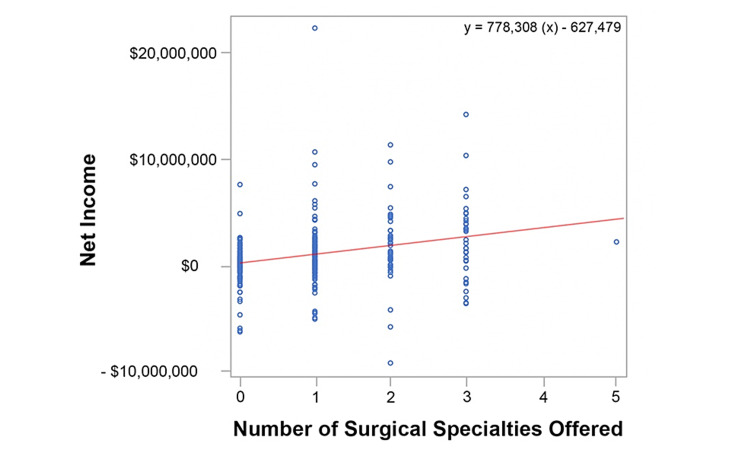
Critical Access Hospital Net Income Per Surgical Specialty Offered (Raw Data)

The data from the linear regression model including co-variate analysis of net income are described in Table 4. The raw data depicted in Figure 2 demonstrates a trend line slope of 778,308 indicating that for each surgical service offered, the net income increases by $778,308. However, the linear regression model including co-variate analysis demonstrates that for every additional surgical service offered, the net income increases by $630,528 (p=0.0032) (Table 4). 

**Table 4:** Critical Access Hospital Net Income Per Surgical Specialty Offered Per unit of surgical services offered, net income increased by $630,528 (p=0.0032). Net income per surgical specialty offered was also analyzed for each of the co-variates. The “Intercept” parameter is the predicted value for the model when all other parameters are set to zero. The column titled “Parameter Estimate” demonstrated the change in the dependent variable according to every one-unit change per predictor variable while all other predictors are kept constant.

Parameter	Parameter Estimate	Standard Error	P-value
Intercept	-867,084	1,225,606	
Number of Surgical Services	630,528	212,294	0.0032
Region (MW vs W)	348,574	462,671	0.8915
Region (NE vs W)	263,872	774,440	0.8915
Region (S vs W)	329,518	497,204	0.8915
Occupancy Rate	15,500	8,111	0.0568
Case Mix Index (CMI)	360,443	1,111,362	0.7457

**Figure 3:**
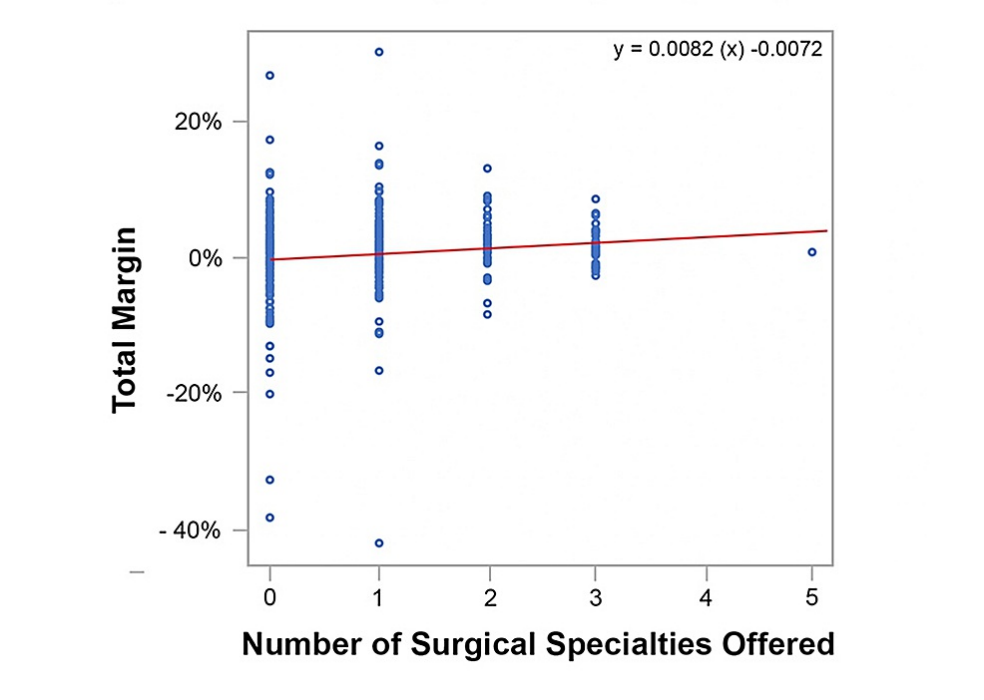
Critical Access Hospital Total Margin per Surgical Specialty Offered (Raw Data)

The data from the linear regression model including co-variate analysis of total margin are described in Table 5. The raw data graphed in Figure 3 demonstrates a trend line slope of 0.0082, indicating that for each additional service offered, the total margin increases by 0.82%. However, the linear regression model including co-variate analysis demonstrates that for every additional surgical service offered, the total margin increases by 0.73%. Of note, this was not a statistically significant finding (p=0.1342) (Table 5). Furthermore, within the raw data, there were no net losses greater than 10% among CAHs equipped with two or three surgical services. None of the CAHs offering three surgical specialties suffered net losses exceeding 2.5%.

**Table 5:** Critical Access Hospital Total Margin per Surgical Specialty Offered Per unit of surgical services offered, total margin increased 0.0073 (p=0.1342). Total margin per surgical specialty offered was also analyzed for each of the co-variates. The “Intercept” parameter is the predicted value for the model when all other parameters are set to zero. The column titled “Parameter Estimate” demonstrated the change in the dependent variable according to every one-unit change per predictor variable while all other predictors are kept constant.

Parameter	Parameter Estimate	Standard Error	P-value
Intercept	0.0152	0.0282	0.5895
Number of Surgical Services	0.0073	0.0049	0.1342
Region (MW vs W)	0.0044	0.0106	0.6825
Region (NE vs W)	0.0177	0.0178	0.3206
Region (S vs W)	-0.005	0.0114	0.6616
Occupancy Rate	0.0001	0.0002	0.5663
Case Mix Index (CMI)	-0.0164	0.256	0.5205

## Discussion

Interpretation of results

The linear regression model for this study demonstrated that for every additional surgical service provided by a CAH, the hospital profits increased by $630,528 (p=0.0032). A similar trend was observed when modeling profitability. Total margin increased 0.73% for each additional surgical service added (p=0.1342). These figures indicate that increasing the number of surgical services offered at a CAH may result in an increase in both net income and total margin. Total margin represents profit as a percentage rather than a gross figure. Total margin can therefore be used to analyze a hospital’s profitability more clearly without the confounder of hospital size.

The slopes found in Figure 2 and Figure 3 differed between the raw data analysis and the linear regression model. The increase in net income and total margin were both less in the linear regression model when compared to the raw data analysis. For example, the raw data depicted in Figure 2 indicated that for each surgical service offered, the net income increased by $778,308. However, the linear regression model (Table 4) demonstrated that the net income increased by $630,528 (p=0.0032). This resulted in a difference of $147,780 in net income.

The raw data graphed in Figure 3 demonstrated the total margin increased 0.82% per surgical service offered. While not statistically significant (p=0.1342), the linear regression model demonstrated that the total margin increased 0.73% (Table 5). This resulted in a difference of 0.09% in total margin. While individually none of the co-variates demonstrated statistical significance, the differences between the two slopes may be attributed to the collective influence of the co-variates. 

There is a preponderance of influences affecting CAH finances, and although surgeries are a key driver of hospital margins, they are but one piece of the puzzle. Therefore, co-variates were used to strengthen the external validity of this study. The co-variates utilized were selected based on availability within the AHD. Occupancy rate may have exerted a minimal effect on the CAHs net income, although this only came close to a statistically significant finding (p=0.0568). This is depicted in Table 4 which demonstrates that a 1% increase in occupancy rate correlated with only a $15,500 increase in net income. Neither geographic region nor CMI displayed a statistically significant effect on either dependent variable. 

The raw data in Figure 3 demonstrated that the total margin of CAHs decreased in variability as the number of surgical services increased. That is, the data points from the CAHs offering zero surgical services showed great variance from mean compared to data from CAHs offering three surgical services. This indicates that CAH profitability seems to become more predictable as more surgical services are offered. Moreover, there was a positive correlation between the number of surgical services and both net income and total margin (Figure 2 and Figure 3, respectively). This finding could be useful for CAHs desiring a more predictable bottom line.

Limitations

A few limitations exist in this study. While the AHD provided adequate financial data for this study, it is not an all-inclusive resource. For instance, the AHD data set provided only three suitable co-variates: CMI, occupancy rate, and geographic region. Furthermore, many CAHs within this study were affiliated with a larger hospital system which may provide financial support to the smaller CAH. CAHs with more financial support may be able to accommodate a higher surgical volume and more complex procedures. However, these affiliations were not always delineated in the AHD. There are undoubtedly many more factors influencing CAH profitability that could not be controlled for using the available data. Further confounders include, but are not limited to, the proximity of competition, population density in the surrounding areas, and quality of medical care. None of these were listed in the AHD, thus could not be controlled for. The inclusion of further covariates would likely strengthen the regression model’s predictive value. Additionally, a one-tailed hypothesis may be merited in future studies to increase power and possibly yield greater significance, given that significance was observed in only one of the two tested variables.

This study lacked data on the surgical capabilities of the physicians caring for patients at CAHs, which could impact some of the findings. If some general surgeons are only performing colonoscopies while others are performing full-spectrum general surgery this could skew the data. A new analysis could be performed based on specific procedures or separated by elective versus emergency surgical services. Another important limitation of this study is that obstetric and gynecologic surgeries were not found in the AHD database for any CAHs utilized in the study, despite some of the sampled CAHs having a labor and delivery unit. The cesarean section is the most common surgery in America; therefore, the surgeries performed at each CAH may be much higher than indicated in the AHD data [9]. These data could be collected from surveys of hospital administrators in future studies.

Any time a sample is utilized in a study, it leaves the potential for discrepancies in the findings. While statistically 300 CAHs adequately samples the population of 1350 CAHs in the United States, the significance level of 0.05 leaves a 5% margin for inaccurate conclusions to be drawn. A final limitation of this study is that these data were manually entered into an Excel spreadsheet leaving room for human error during the data entry process.

Applications of findings

The current findings illuminate an existing trend in which surgical services augment the profits of CAHs. It follows that the inverse is also true: not having a surgery department can be considered an economic disadvantage. This study confirms what the literature suggests about the relationship between surgical services and hospital profits, but specifically at CAHs. This study provides compelling evidence to increase the number of surgical services at CAHs.

Furthermore, the economic health of CAHs impacts the health of rural Americans, which includes almost 20% of the United States population [19]. The development of surgical services at CAHs could influence the success of local healthcare delivery by improving the net income of CAHs; increasing surgical services at CAHs may impact the hospital’s viability. If CAHs can remain operational, this should improve access to medical and surgical care for rural communities.

Future directions

Analysis of more co-variates could broaden the applications of this study. For example, partnership with healthcare systems is a co-variate that could help to financially support CAHs [10]. These partnerships are not uncommon: in a sample spanning from 2005 to 2012, 8.8% of 1,492 rural hospitals experienced an acquisition or merger. Additional studies could attempt to further stratify the data to better control for certain hospital characteristics such as size and Accountable Care Organization (ACO) affiliation. This may allow comparisons between partnered hospitals and hospitals which are not affiliated with a larger facility or an ACO. Additional co-variates that could be studied include, but are not limited to, the proximity of competition, population density in the surrounding areas, and various quality metrics.

Further research could analyze the impact of surgical networking on CAH viability. For example, some surgeons have independently strategized to keep their local CAHs staffed with surgical services. In Montana, Dr. David Sheldon has established a community of surgeons with excellent networking and patient-focused care [20]. Here, rather than outsourcing patient care or forcing the use of locum tenens services, a program known as the Rural Surgery Support System (RS3) has been established. RS3 helps surgeons collaborate and overcome constraints to keep patients at home by supporting each other directly. The more experienced surgeons will come to the aid of other surgeons for complex cases. This system not only increases patient access to surgical services but also provides increased financial security by continuing to allow surgical services to be offered at CAHs. However, whether this sort of strategy would prove beneficial to the entirety of the U.S. remains in question. Despite this, there is potential for implementing a similar program on a larger scale within the United States, to determine if other CAHs could benefit from a similar model. This could improve access to surgical care at CAHs and theoretically improve CAH viability.

Rural hospitals know the benefit of surgical services but have difficulty recruiting surgeons due to a lack of funding and decreased surgical workforce in rural areas. These hospitals often resort to the use of locum tenens surgeons when unable to find local surgeons, which may lead to worse outcomes [21]. The rural healthcare workforce is vastly augmented by the National Health Service Corps (NHSC), an organization that pays off the student loans of primary care, mental health, and dental health workers who opt to practice for a time in a rural municipality. Currently, surgeons do not currently qualify for this financial opportunity, despite the known shortage of surgeons serving rural areas [21-23]. For hospitals, recruiting surgeons to rural employment is complicated by a lack of financial bargaining power as well as limited academic opportunity [23,24]. If the NHSC program were expanded to include surgeons, it could ease the costs of starting and maintaining surgery programs among CAHs and improve rural surgeon recruitment. Further studies could be done to analyze the benefit of surgical recruitment programs for CAHs and if it impacts the success of the facility.

Recommendations

The following recommendations are based on this study’s findings:

1. This study demonstrates that there is a positive correlation between the number of surgical services offered and CAH net income. Furthermore, the profitability of CAHs offering multiple surgical specialties seems more stable and predictable than the profitability of CAHs offering none. It is worth noting that this study’s co-variates represent factors that hospitals have virtually no control over. Hospitals do have a degree of choice when it comes to the directions in which they expand their operations. Therefore, it is recommended that CAHs should offer new surgical services or expand existing ones to improve net income.

2. Additional studies should be performed to analyze the impact of additional co-variates on this study’s findings. These potential co-variates include but are not limited to hospital system affiliation status, ACO affiliation status, hospital size, the proximity of competition, population density in the surrounding areas, and quality of medical care provided. These data could be collected via surveys from hospital administrators and could also include data about obstetric and gynecologic surgeries performed at CAHs.

## Conclusions

CAHs play an essential role in ensuring access to health care for individuals living in rural areas in the United States. Rural hospitals face unique financial adversities. Net income among CAHs is significantly correlated to the number of surgical services offered by a hospital. Although total margin analysis yielded no significant results, hospitals providing three surgical services demonstrated less variance from the mean, and thus more predictable profitability, than those offering less. These findings indicate that CAHs could financially benefit from offering new surgical services or expanding existing ones.
